# Application of multimodal imaging in the diagnosis of intrahepatic splenosis: Two case reports and a literature review

**DOI:** 10.1259/bjrcr.20210170

**Published:** 2022-01-10

**Authors:** Weimin Liu, Simin Chen, Jianning Chen, Ting Jiang, Li Quan, Sidong Xie

**Affiliations:** 1 Department of Radiology, The Third Affiliated Hospital of Sun Yat-sen University, Guangzhou, China; 2 Department of Radiology, Zhao Qing City Gao Yao District People’s Hospital, Zhaoqing, China; 3 Department of Pathology, The Third Affiliated Hospital of Sun Yat-sen University, Guangzhou, China

## Abstract

Intrahepatic splenosis is quite rare and is often misdiagnosed as other lesions. We present two cases of intrahepatic splenosis examined with hepatobiliary contrast agents, intravoxel incoherent motion diffusion-weighted imaging and magnetic resonance elastography. We discuss various imaging modalities and the roles of various magnetic resonance imaging methods in diagnosis. We also discuss the differentiating features that allow the correct diagnosis to be made and provide a brief review of the literature.

## Summary

Spleen implantation is a space-occupying lesion caused by the growth of splenic tissue in organs or tissues other than the spleen; this lesion usually arises after splenic trauma or surgery. Intrahepatic splenosis (IHS) is rare and is difficult to diagnose clinically because its imaging manifestations are similar to the imaging manifestations of other intrahepatic neoplastic lesions. In this report, we retrospectively analysed two clinical cases of IHS confirmed by surgical resection and histopathological examination. We also summarized the clinical imaging characteristics of IHS, especially with the application of liver-specific contrast agents, intravoxel incoherent motion diffusion-weighted imaging (IVIM-DWI) and magnetic resonance elastography (MRE), to provide more references for the diagnosis of this disease, to improve the diagnostic accuracy of this disease and to reduce unnecessary surgical exploration.

## Case one

A 37-year-old male was admitted to our hospital for further investigation of an intrahepatic mass found during a routine examination. The patient claimed to experience no discomfort and had a history of posttraumatic splenectomy 24 years prior to admission. The routine blood and liver–renal function laboratory test results remained normal, and the serological biomarkers of hepatitis B virus (HBV) and hepatitis C virus (HCV) infections were negative. The serum levels of tumour biomarkers [alpha-fetoprotein (AFP), carcinoembryonic antigen (CEA), CA-199, CA-125, and CA-153] were within the normal range.

**Figure 1.**
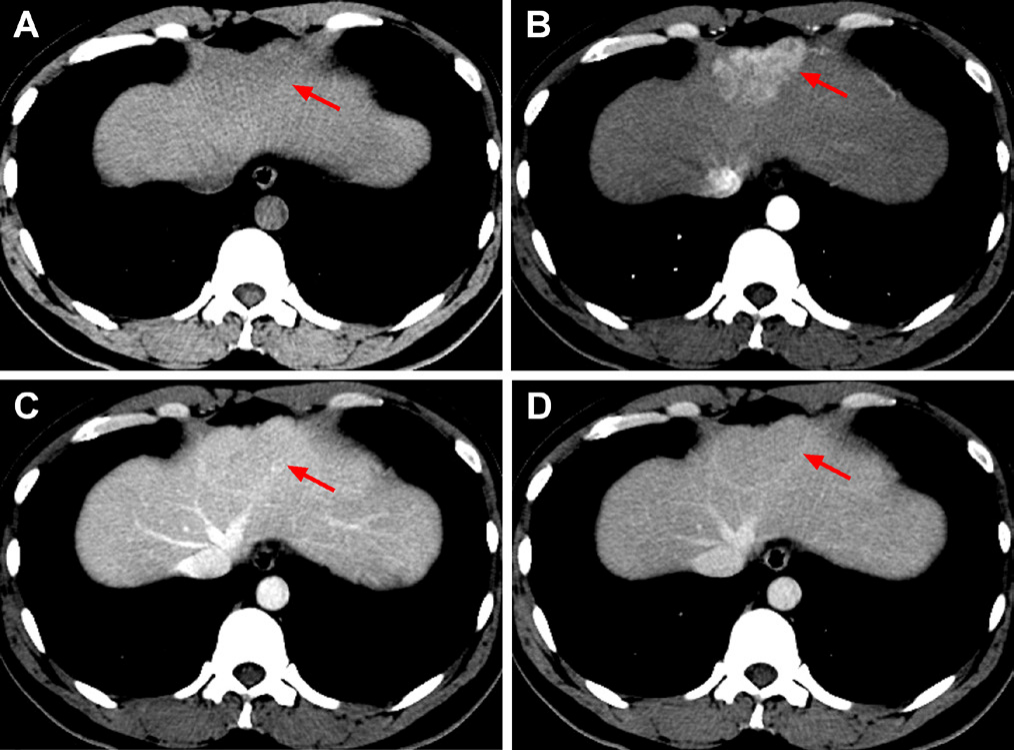
CT images of IHS in case one. Non-contrast CT revealed a round, slightly hypodense mass located in segment II/IV of the left lobe of the liver with an unclear boundary (A, arrow). After contrast injection, the mass exhibited heterogeneous hyperenhancement in the arterial phase (B, arrow), isodensity in the portal venous phase (C, arrow), slight hypodensity in the delayed phase (D, arrow) and delayed enhancement of the capsule (C, D, arrow).

Computed tomography (CT) scanning revealed a slightly hypodense mass located in segment II/IV of the left lobe of the liver, and the mass measured 5.6 × 3.7 cm and had an unclear boundary. After contrast injection, the lesion showed heterogeneous hyperenhancement in the arterial phase, isodensity in the portal venous phase, slight hypodensity in the delayed phase and delayed enhancement of the capsule (Figure 1). The spleen was not seen on CT. A diagnosis of focal nodular hyperplasia (FNH) was made based on the CT findings.

**Figure 2.**
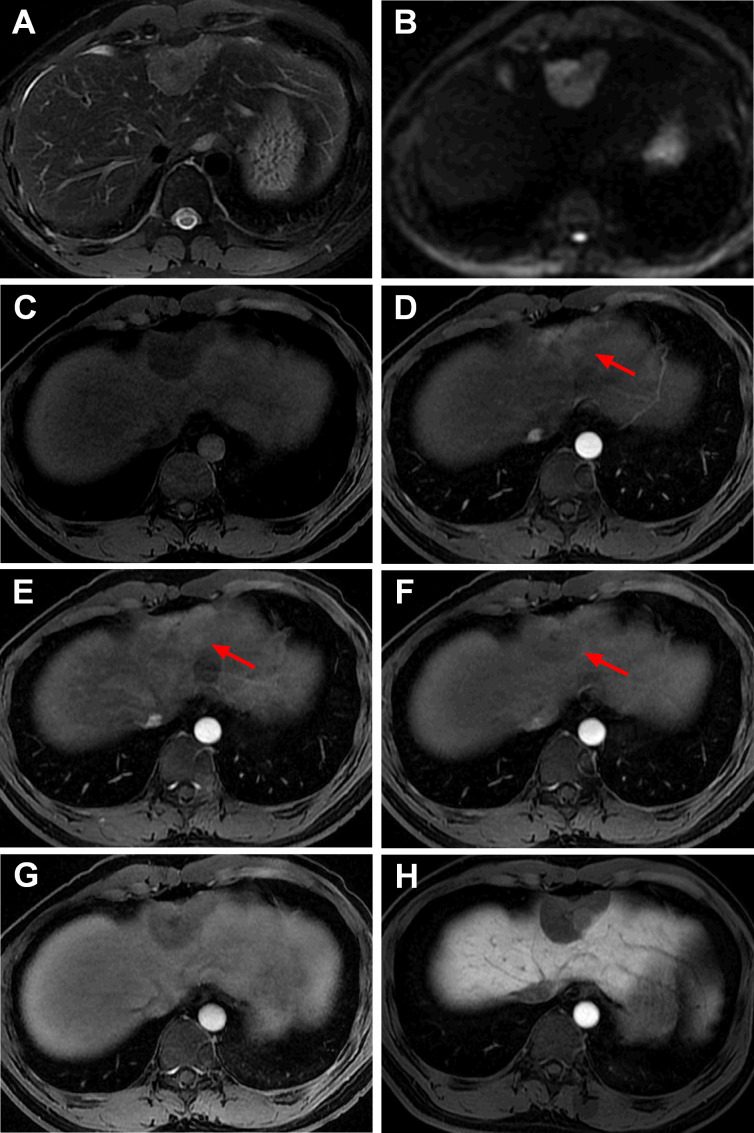
MRI images of IHS in case one. The segment II/IV mass was slightly hyperintense on the *T*_2_-weighted images (**A**), hyperintense on the diffusion-weighted images (**B**), and hypointense on the *T*_1_-weighted images (**C**). After injection of Gd-EOB-DTPA, the mass appeared heterogeneous hyperintense during the early and late arterial phases (D, E, arrow), relatively slightly hypointense in the portal vein phase (F, arrow), hypointense in the transitional phase (**G**), and obviously hypointense in the hepatobiliary phase (**H**).

**Figure 3.**
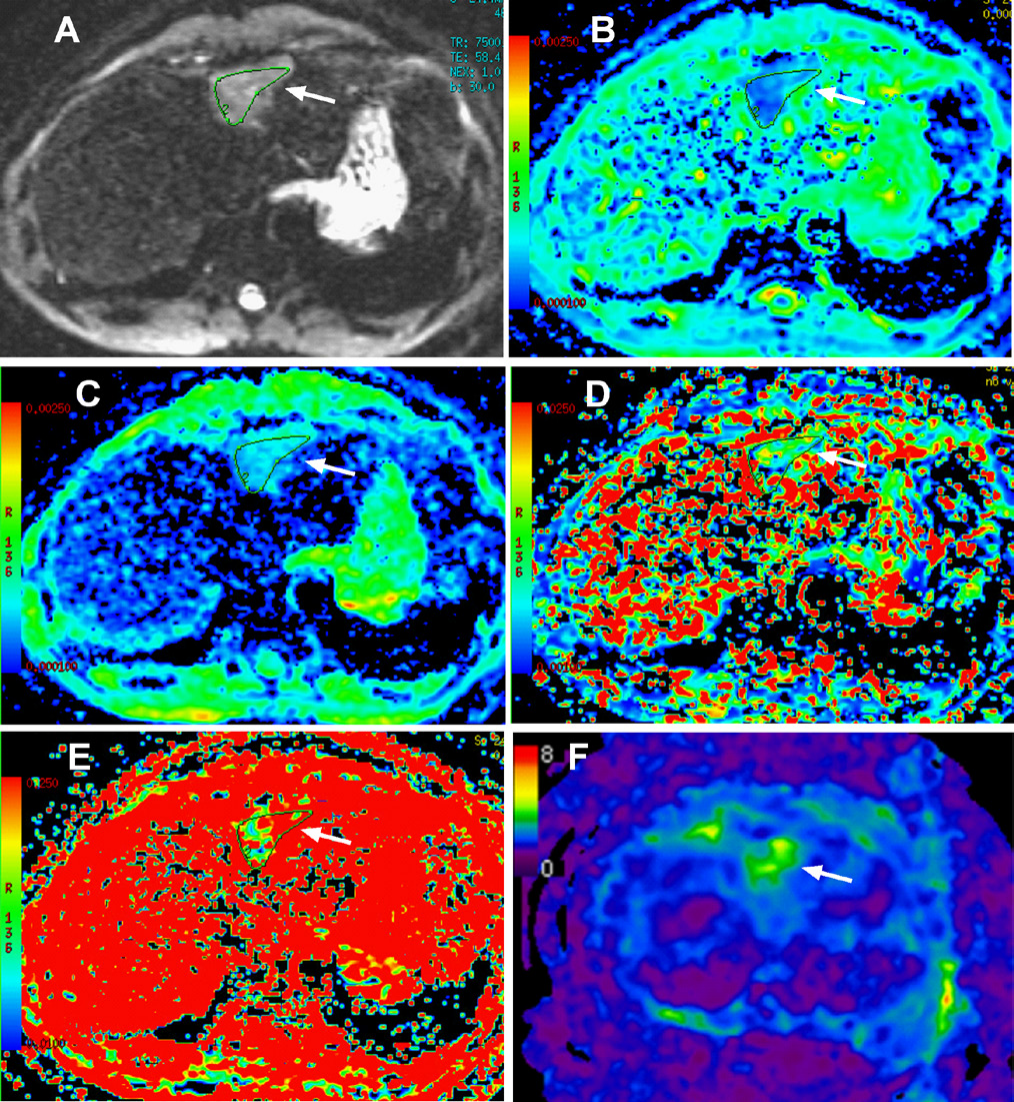
Intravoxel incoherent motion diffusion-weighted imaging (IVIM-DWI) and magnetic resonance elastography (MRE) of IHS in case one. The segment II/IV mass was hyperintense on DWI (A, *b* = 0 s/mm^2^, arrow); compared with those of the adjacent normal liver parenchyma, the *ADC_std_* (**B, arrow**), *ADC_fast_* (**D, arrow**) and *f* (**E, arrow**) of the mass were decreased, and the *ADC_slow_* (**C, arrow**) was increased. The stiffness value of IHS was 3.7 kPa on MRE, higher than the surrounding normal liver parenchyma (**F, arrow**).

Magnetic resonance imaging (MRI) showed that the segment II/IV mass was hypointense on *T*_1_-weighted imaging (T1WI), slightly hyperintense on *T*_2_-weighted imaging (T2WI) and hyperintense diffusion-weighted imaging (DWI). After the injection of gadolinium ethoxybenzyl dimeglumine (Gd-EOB-DTPA) (Bayer Healthcare, Berlin, Germany), the mass appeared heterogeneously hyperintense during the early and late arterial phases, relatively slightly hypointense in the portal vein phase, hypointense in the transitional phase, and obviously hypointense in the hepatobiliary phase (Figure 2). Moreover, IVIM-DWI and 60 Hz 3D-MRE examinations were performed in this patient. Compared to those of the surrounding liver parenchyma, the *ADC_std_*, *ADC_fast_* and *f* of the mass were decreased, and the *ADC_slow_* was increased (Figure 3A–E). The stiffness value of the mass in 3D-MRE was 3.7 kPa, while the surrounding normal liver parenchyma was 1.8 kPa (Figure 3F). A diagnosis of atypical FNH was made based on MRI, but a hepatic adenoma or a low-grade malignant tumour could not be ruled out.

**Figure 4.**
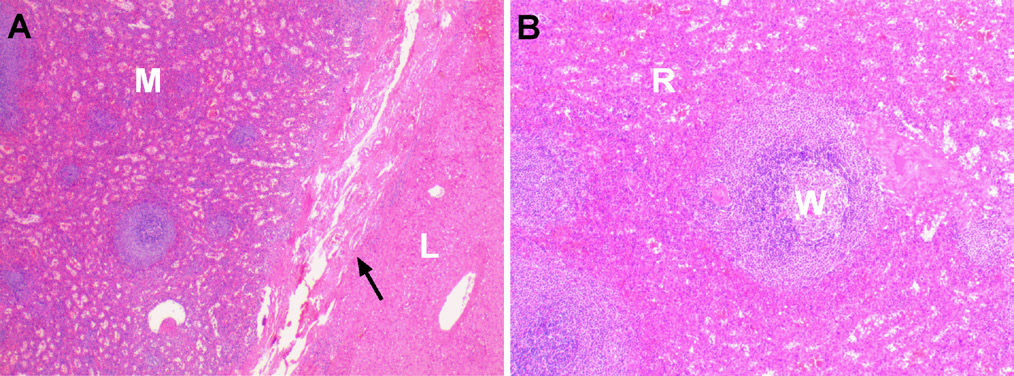
Pathological images of IHS in case one. H&E staining (A, low power field, 40×) shows that the splenic tissues on the left and the liver tissues on the right are clearly demarcated by thick fibrous capsules (arrow). The hepatic lesion was composed of white pulp and red pulp (B, low-power field, 100×), consistent with splenosis. (L, liver; M, mass; R, red pulp; W, white pulp; arrow, fibrous capsule).

A partial hepatectomy was performed because the imaging examinations did not rule out malignant tumours. During surgery, abdominal exploration revealed that the mass was located in segment II/IV of the left lobe of the liver. It was greyish red, exogenous, adhered to the peritoneum, and had an intact capsule, clear boundary and soft texture. The spleen was absent, and the mass was completely removed during the surgery. Histopathology, including postoperative haematoxylin and eosin (HE) staining, revealed that the mass consisted of splenic tissue, there was a capsule that separated the splenic tissue from the liver tissue (Figure 4), and the diagnosis of IHS was confirmed. The patient was discharged uneventfully after the operation, and no recurrence was observed during the 2 years of follow-up.

## Case two

A 39-year-old male was admitted to the hospital with an intrahepatic lesion found on an abdominal ultrasound examination. He underwent splenectomy in 2002 due to a car accident. No significant signs were observed during the physical examination, and no obvious abnormalities were observed in the routine blood and biochemical examinations. Moreover, the patient’s serological analyses for HBV or HCV infections were negative.

**Figure 5.**
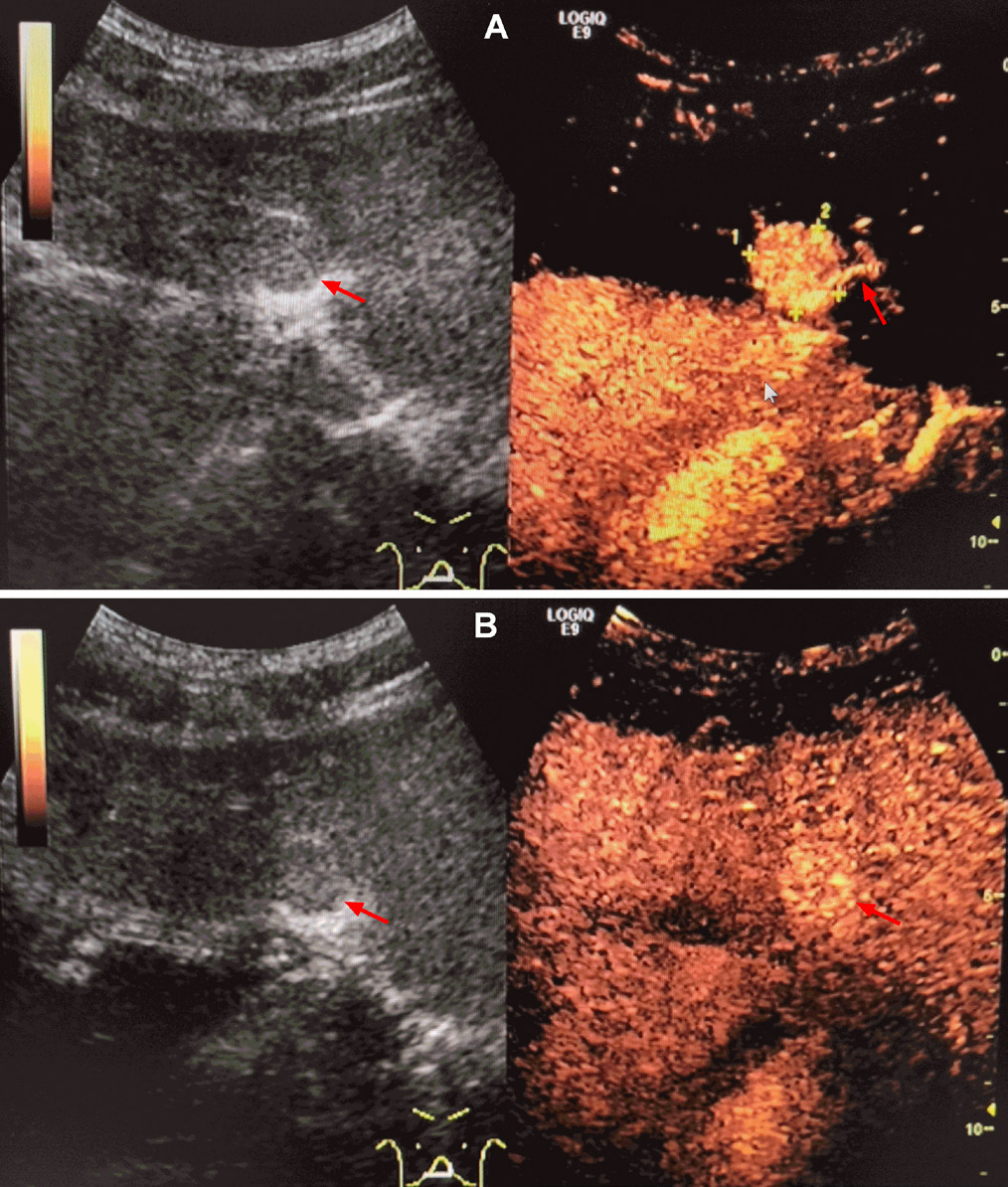
Ultrasound images of IHS in case two. The lesion in segment II was a homogenous isoechoic nodule (A, arrow) and had a size of 2.0 × 1.8 cm with an unclear boundary. Contrast-enhanced ultrasonography showed that the lesion exhibited a high enhancement in the arterial phase with a large vessel entering the lesion (A, arrow) and a continued slightly high enhancement in the sinusoidal phase (B, arrow).

Abdominal ultrasound showed that a slightly circular isoechoic lesion in the left hepatic outer lobe had a size of 2.0 × 1.8 cm with unclear boundaries, and the internal echotexture was relatively homogenous. Contrast-enhanced ultrasonography (CEUS) with Sonovue (Bracco, Milan, Italy) showed that the lesion had a high enhancement in the arterial phase, with a large vessel entering the lesion, and a continued slightly high enhancement in the portal vein phase and the sinusoidal phase (Figure 5).

**Figure 6.**
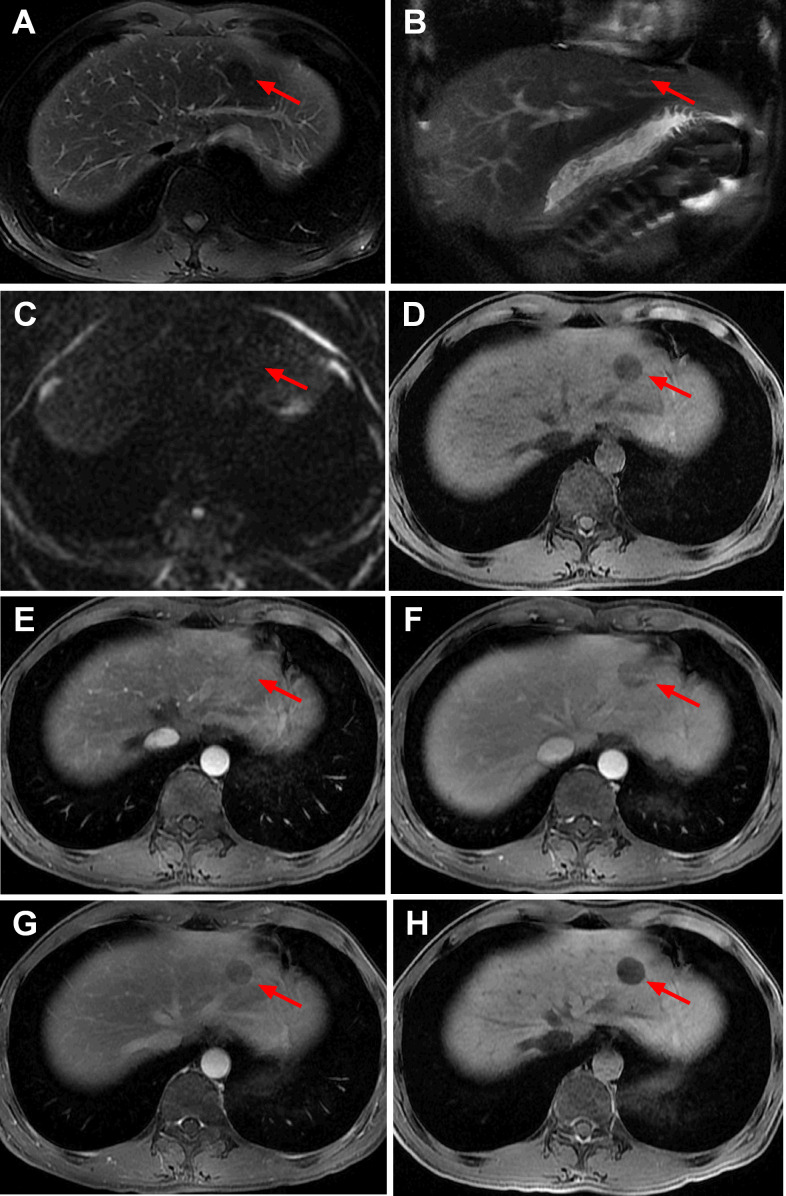
MRI images of IHS in case two. The lesion in segment II had a low signal on T2WI-FS (A, arrow) and was located in the peritoneal folding area between the diaphragm, falciform ligament, and left hepatic capsule (B, arrow). The lesion was relatively isointense on DWI (C, arrow) and hypointense on T1WI (D, arrow) with a clear boundary. After the injection of Gd-BOPTA, the lesion exhibited isointense enhancement in the arterial phase (E, arrow), hypointensity in the portal vein phase (F, arrow) and delayed phase (G, arrow), and an enhancement defect in the hepatobiliary phase after a delay of 2 h (H, arrow).

**Figure 7.**
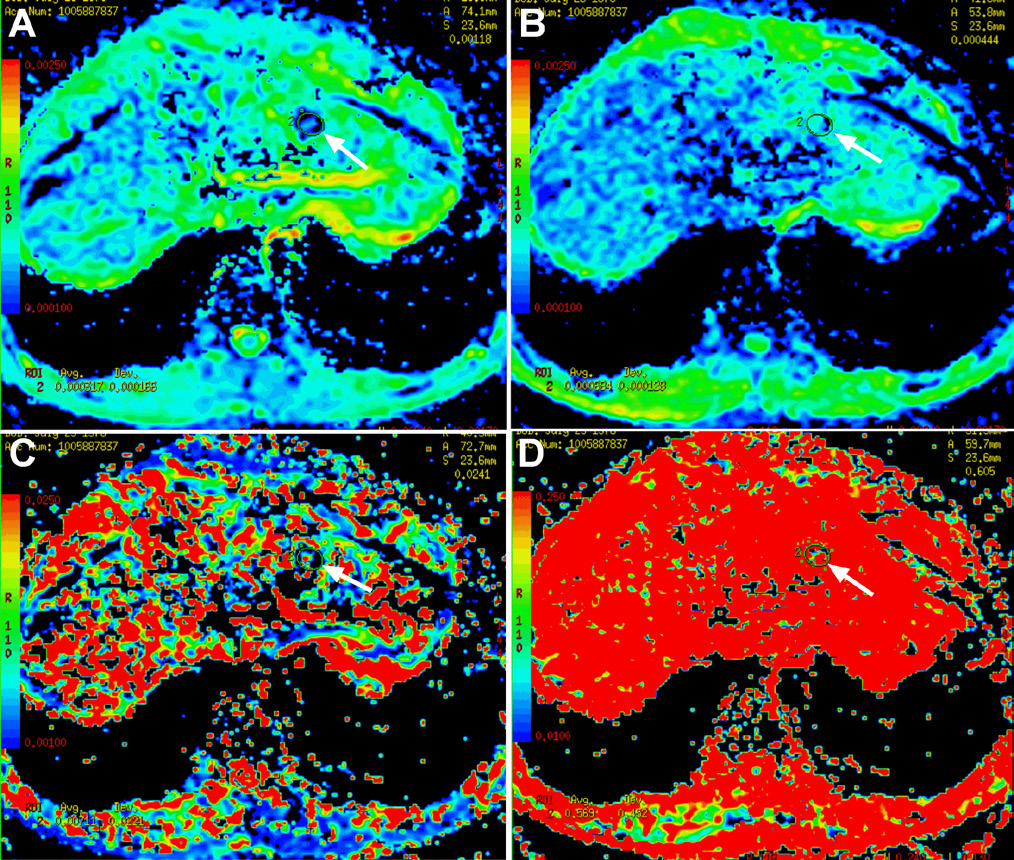
Intravoxel incoherent motion diffusion-weighted imaging (IVIM-DWI) of IHS in case two. Compared with those of the adjacent normal liver parenchyma, the *ADC_std_* (A, arrow), *ADC_slow_* (B, arrow), *ADC_fast_* (C, arrow) and *f* (D, arrow) of the lesion in segment Ⅱ were all reduced.

MRI showed that the hepatic nodule in segment II was hypointense on T1WI and T2WI and had clear boundaries and a size of approximately 2.1 × 1.7 cm. Dynamic-enhanced MR scanning with gadobenate dimeglumine (Gd-BOPTA) (Bracco, Shanghai, China) showed that the lesion exhibited isointense enhancement in the arterial phase, hypointensity in the portal vein phase and delayed phase, and an enhancement defect in the hepatobiliary phase after a delay of 2 h (Figure 6). The mass was relatively isointense on DWI. The IDEAL IQ sequence showed that the R2* value in the lesion was 228 ~ 274. Moreover, an IVIM-DWI examination was performed and compared with those of the adjacent normal liver parenchyma, the *ADC_std_, ADC_slow_*, *ADC_fast_* and *f* of the lesion were all reduced (Figure 7). A diagnosis of segment II hepatic benign nodule with iron overload was made based on MRI, and the differential diagnosis included atypical neoplasm or IHS.

**Figure 8.**
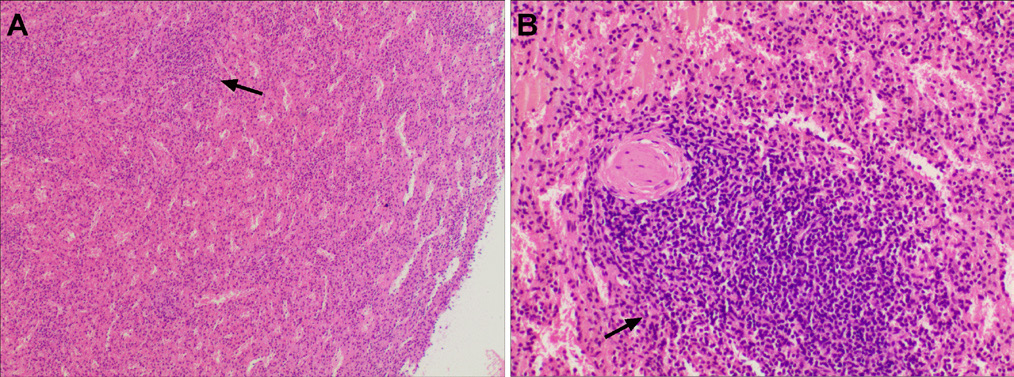
Histopathological features of IHS in case two. H&E staining showed that the lesion was composed of white pulp (arrow) and red pulp (A, low power field, 100×; B, middle power field, 200×).

Based on the imaging findings alone, tumours could not be excluded, and the patient subsequently underwent a left lateral segmentectomy. The surgical findings showed that there was a dark red nodule located between the diaphragm and the hepatic left lateral lobe, and the nodule had a smooth surface, a soft texture and an intact capsule. The lesion was embedded within the left hepatic lateral lobe. Histopathology showed that red pulp and white pulp structures could be seen in the lesion under a microscope, and the histopathological diagnosis of IHS was established (Figure 8).

## Discussion

IHS is extremely rare, with fewer than 60 cases published to date.^1–3^ Non-invasive diagnosis of IHS is often challenging since it is prone to mimicking other liver tumours or neoplastic lesions. IHS is usually an incidental asymptomatic finding. According to a review of previous literature reports, almost all his patients, except two, had a history of splenectomy.^1,4,5^ Furthermore, the main reason for splenectomy was traumatic rupture of the spleen. The above two cases were consistent with literature reports.

According to the literature review,^1,6–8^ IHS usually presents as single or multiple lesions at the surface of the liver or within the liver parenchyma, especially between the diaphragm, falciform ligament, and left hepatic capsule. In our two cases, IHS was described as invagination of the peritoneal folding area between the diaphragm, falciform ligament, and left hepatic capsule, which involved almost the same location as the literature reports.^9–12^ This peritoneal folding area was considered the specific hepatic splenosis site and an important imaging feature for IHS.^9^ The mechanism underlying this phenomenon may be the direct dissemination of splenic tissue in the peritoneal folding area, and the chronic effect of compressing the diaphragm easily leads to the invasion of the splenic tissue into the liver parenchyma.^13^

The characteristic imaging of IHS on CT findings includes lesions that are hypodense on non-contrast CT, hyperdense in the arterial phase, isodense in the portal venous phase, and hypodense in the delayed phase.^1,11^ The CT findings of case one of our report are similar to those from previous literature; in addition, the lesion had an enhancement rim in the portal venous phase and delayed phase, which has rarely been reported in the literature.^8,11^ This rim was actually a pseudocapsule. Because the lesion was close to the perihepatic capsule, we speculated that it gradually grew and then eventually compressed the adjacent liver capsule to form a pseudocapsule. The presence of a rim surrounding the lesion has been described as a characteristic finding of IHS.^11^ This rim could show thin hyperechoic areas on ultrasonography, low densities on CT or low signal intensities on T1WI and T2WI, or a delayed enhancement ring, which represents a thin layer of fat or fibrous capsule around the lesion.^5,8,9,11,14,15^

The MRI findings of IHS included homogeneous hypointensity in T1WI and hyperintensity in T2WI.^11^ However, the lesion in case two of our report showed a low signal on T2WI, which is rare in the literature.^16–19^ Although berlin blue staining was not performed on this lesion, the R2* value of the lesion was 228 ~ 274 in the IDEAL-IQ sequence, which is a quantitative indicator of iron deposition,^20^ suggesting iron overload in the lesion. This may be caused by the phagocytosis of iron particles by splenic reticuloendothelial cells and can be confirmed by histology to help support this speculation.^16^ Therefore, intrahepatic lesions that demonstrate either iso- or hypointensity on T2W MRI may also support the diagnosis of IHS.

The two cases in our study were given hepatocyte-specific MR contrast agents that can be taken up specifically by hepatocytes and then excreted by the bile ducts. Hepatocyte-specific contrast agents in these two cases had different manifestations in the arterial phase, presumably due to iron overload in case two. Both cases of IHS in our report showed strong hypointensity in the hepatobiliary phase, which was useful to indicate that the lesions had no hepatocytes, and it can be conducive to differentiating lesions that involve the presence of normal hepatocytes, such as FNH. In the literature, only four studies on IHS that used hepatocyte-specific contrast agents for enhanced scanning have been published.^2,5,15,17^ In clinical practice, the combination of conventional MRI findings and hepatobiliary phase images is very helpful for differentiating intrahepatic lesions.

To the best of our knowledge, we are the first report the use of MRE for the diagnosis of IHS. In case one, the lesion (3.7 kPa) had a higher tissue stiffness than the healthy liver (1.8 kPa). Several studies have shown that the stiffnesses of healthy liver, benign liver tumours or malignant liver tumours were 2.7 ± 0.4 kPa, 2.3 ± 0.3 kPa or 10.1 ± 3.6 kPa, respectively.^21–23^ Based on the reference values for tissue stiffness, the lesion in our study should be a benign mass. Mannelli et al^24^ reported that normal spleen stiffness is not significantly correlated with age, body mass index, arterial mean blood pressure, spleen volume or liver stiffness. The reported normal spleen stiffness can vary from 2.35 to 5 kPa, with a mean value of 3.6 kPa.^24,25^ Therefore, the IHS in case one had a stiffness value similar to that of a healthy spleen. We believe that the stiffness value of MRE is helpful to distinguish IHS from hepatic malignant tumours, but this speculation needs to be confirmed by more research in the future.

According to the review of previous literature, only four cases reported the manifestations of IHS on the DWI sequence^26–29^ ; all of these studies showed relatively high signals on DWI, and one study showed a signal reduction in the ADC images. In the present cases, IHS also had a high signal on DWI and a decreased ADC value, which was consistent with the previous literature and similar to the performance of the normal spleen. However, there were no signal differences on DWI between the IHS and the nearby hepatic tissue in case two, which was hypothesised to be the result of iron overload.

In addition, IVIM-DWI was performed in these present two patients, and this has not been reported in the previous literature. The *ADC_std_* of IHS was lower than that of the adjacent liver tissue, while the *ADC_fast_* and *f* of the perfusion-related coefficient were also decreased. This indicates that the degree of restricted IHS diffusion was higher than that in the liver, and the blood perfusion was lower than that in the liver. This may be because IHS is supplied by surrounding vessels.^11,27^ IVIM-DWI could be helpful when the gadolinium dose is decreased or not given due to contraindications in renal failure patients.

The total number of case reports about IHS with CEUS examination is small,^2,3,30,31^ and CEUS imaging of IHS includes variable arterial phase enhancement and sustained enhancement throughout the portal and sinusoidal phases. Based on the pharmacological kinetics in the SonoVue contrast study,^32^ Lim et al thought that SonoVue may be a spleen-specific enhancement agent because this contrast agent remains in the spleen for up to 5 min. This characteristic CEUS finding has a specific value for the diagnosis of IHS, and it needs to be further verified in a larger sample size.

In general, the imaging features of IHS are not characteristic; therefore, differential diagnosis is very important. Since IHS usually has a rich blood supply, it is necessary to distinguish IHS from other hypervascularised lesions, such as HCC, FNH and hepatic adenoma. For hepatic lesions in patients with high-risk factors for HCC, the differentiation between HCC and IHS is very important. According to a recent literature review (which summarised 59 patients with IHS during the period of 1939 to 2019),^1^ 17 patients had HBV and/or cirrhosis of high-risk factors for HCC, and 14 of these patients were initially misdiagnosed with HCC. Therefore, it is very important to exclude HCC in this group of patients. Combining factors such as normal AFP, the history of traumatic splenectomy, peritoneal folding of specific hepatic splenosis sites, lack of intralesional fat, lack of intralesional necrosis, lack of signs of malignant invasion, lack of obvious washout, strong hypointensity in the hepatobiliary phase with hepatocyte-specific MR contrast agents, similar normal spleen stiffness value on MRE, etc., it is useful to exclusion of the possibility of HCC. At this point, Tc-99m heat-damaged RBC scintigraphy examination is a non-invasive, specific and relatively sensitive method to confirm the diagnosis of splenosis,^33^ as the spleen contains more than 90% of heat-damaged RBCs.^34,35^ However, the improper preparation of heat-damaged RBCs, such as with overheating or underheating, may result in false negative results,^36^ and Toh et al^1^ showed that Tc-99 m-labelled heat-damaged RBCs were not widely used to diagnose IHS, possibly due to their limited availability or cost.

Previous literature has reported that superparamagnetic iron oxide (SPIO)-MRI is helpful for differentiating HCC from IHS. IHS often exhibits hypointensity on *T*_2_-weighted MRI images due to the phagocytosis of iron particles by splenic reticuloendothelial cells, while HCC (without reticuloendothelial cells) is relatively hyperintense on T2WI.^37^ However, IHS showed only a 50% loss in the signal intensity and remained slightly hyperintense relative to the hypointense liver parenchyma^16,37^ ; these findings might not be helpful for the diagnosis. Furthermore, SPIO-MRI is not currently available for use in humans worldwide.

If the patient does not have a high risk for HCC, IHS should be distinguished from intrahepatic benign lesions, such as FNH, hepatic adenoma, and haemangioma. FNH on MRI appears almost as an isointensity on T1WI, T2WI and DWI, and the central scar of FNH shows a low signal on T1WI, a high signal on T2WI, and delayed enhancement. In addition, the key imaging findings for the differentiation of FNH from IHS are a high/iso signal intensity on HBP with hepatobiliary contrast agents. Hepatic adenoma is more common in young females, usually contains fatty degeneration and capsular signs, and often presents with concurrent bleeding and necrosis. These manifestations are helpful for the differential diagnosis of IHS.

In conclusion, IHS is rare, and patients usually have a history of traumatic splenic rupture and splenectomy, incidental asymptomatic findings, and normal laboratory examination. IHS mainly occurred near the hepatic capsule, especially the peritoneal folding area between the diaphragm, falciform ligament, and left hepatic capsule. The imaging findings include variable arterial phase enhancement, sustained enhancement or washout in the portal venous phase and delayed phase, a pseudocapsule rim sign, strong hypointensity in the hepatobiliary phase, relatively high signal on DWI or IVIM-DWI, similar normal spleen stiffness value on MRE, and sometimes iron overload. Combining US, CT, MRI (including the use of hepatobiliary contrast agents), IVIM, MRE and other imaging methods, including Tc-99m heat-damaged RBC scintigraphy, these multimodal imaging technologies are helpful for the non-invasive diagnosis and differential diagnosis of IHS.

## Learning points

1. In the case of hepatic lesions with incidental asymptomatic findings, if the patient had a history of splenectomy due to splenic trauma, no high-risk factors for HCC, and normal laboratory examination, IHS should be considered in the differential diagnosis.
2. IHS often exhibits variable arterial phase enhancement, sustained enhancement throughout the portal and sinusoidal phases on CEUS, sustained enhancement or washout in the portal venous phase and delayed phase of CE-CT/MRI, and strong hypointensity in the hepatobiliary phase with hepatocyte-specific MR contrast agents.
3. IHS mainly occurs near the hepatic capsule, especially the peritoneal folding area between the diaphragm, falciform ligament, and left hepatic capsule, often has a pseudocapsule rim, and sometimes exhibits iron overload.
4. IHS has stiffness values similar to those of normal spleen tissue on MRE, which may be helpful to distinguish IHS from HCC.
5. IHS often shows a relatively high signal on DWI, the *ADC_std_* of IHS is lower than that of the adjacent liver tissue, and the *ADC_fast_* and *f* are also decreased on IVIM-DWI.

## References

[1] Toh WS, Chan KS, Ding CSL, Tan CH, Shelat VG. Intrahepatic splenosis: a world review. *Clin Exp Hepatol* 2020; **6**: 185–98. doi: 10.5114/ceh.2020.99509 33145425 PMC7592095

[2] Sansone V, Falsetti L, Tovoli F, Golfieri R, Cescon M, Piscaglia F. An uncommon focal liver lesion: intrahepatic splenosis. *J Gastrointestin Liver Dis* 3, 2020; **29**: 257–62. doi: 10.15403/jgld-617 32530993

[3] Zhong X, Yang L, Huang J, Deng L, Nie L, Lu Q. Contrast-enhanced ultrasonographic imaging of hepatic splenosis: a case report. *Medicine (Baltimore*) 22, 2021; **100**(3): e24243. doi: 10.1097/MD.0000000000024243 33546044 PMC7837972

[4] Mescoli C, Castoro C, Sergio A, Ruol A, Farinati F, Rugge M. Hepatic spleen nodules (hsn). *Scand J Gastroenterol* 2010; **45**: 628–32. doi: 10.3109/00365521003587812 20408775

[5] Sato N, Abe T, Suzuki N, Waragai M, Teranishi Y, Takano Y, et al. Intrahepatic splenosis in a chronic hepatitis c patient with no history of splenic trauma mimicking hepatocellular carcinoma. *Am J Case Rep* 27, 2014; **15**: 416–20. doi: 10.12659/AJCR.890999 25261602 PMC4179547

[6] Wu C, Zhang B, Chen L, Zhang B, Chen X. Solitary perihepatic splenosis mimicking liver lesion: a case report and literature review. *Medicine (Baltimore*) 2015; **94**(9): e586. doi: 10.1097/MD.0000000000000586 25738479 PMC4553962

[7] Davidson LA, Reid IN. Intrahepatic splenic tissue. *J Clin Pathol* 1997; **50**: 532–33. doi: 10.1136/jcp.50.6.532 9378826 PMC500007

[8] De Vuysere S, Van Steenbergen W, Aerts R, Van Hauwaert H, Van Beckevoort D, Van Hoe L. Intrahepatic splenosis: imaging features. *Abdom Imaging* 2000; **25**: 187–89. doi: 10.1007/s002619910042 10675464

[9] Pekkafali Z, Karsli AF, Silit E, Başekim CC, Narin Y, Mutlu H, et al. Intrahepatic splenosis: a case report. *Eur Radiol* 2002; **12 Suppl 3**: S62-5. doi: 10.1007/s00330-002-1561-5 12522606

[10] Liu K, Liang Y, Liang X, Yu H, Wang Y, Cai X. Laparoscopic resection of isolated hepatic splenosis mimicking liver tumors: case report with a literature review. *Surg Laparosc Endosc Percutan Tech* 2012; **22**: e307-11. doi: 10.1097/SLE.0b013e318263a3f3 23047415

[11] Tsitouridis I, Michaelides M, Sotiriadis C, Arvaniti M. CT and mri of intraperitoneal splenosis. *Diagn Interv Radiol* 2010; **16**: 145–49. doi: 10.4261/1305-3825.DIR.1855-08.1 19838993

[12] Teles GNS, Monteiro PEZ, Raphe R. Intrahepatic splenosis mimicking hepatic neoplasia. *Int J Surg Case Rep* 2018; **44**: 47–50. doi: 10.1016/j.ijscr.2018.02.021 29475171 PMC5927808

[13] Choi G-H, Ju M-K, Kim J-Y, Kang C-M, Kim K-S, Choi J-S, et al. Hepatic splenosis preoperatively diagnosed as hepatocellular carcinoma in a patient with chronic hepatitis b: a case report. *J Korean Med Sci* 2008; **23**: 336–41. doi: 10.3346/jkms.2008.23.2.336 18437023 PMC2526445

[14] Luo X, Zeng J, Wang Y, Min Y, Shen A, Zhang Y, et al. Hepatic splenosis: rare yet important - a case report and literature review. *J Int Med Res* 2019; **47**: 1793–1801. doi: 10.1177/0300060519828901 30810057 PMC6460629

[15] Kawada S, Ichikawa T, Ueda H, Ito K, Inoue K, Mori K. A case of intrahepatic splenosis: usefulness of splenic scintigraphy. *Abdom Radiol (NY*) July 2020; **45**: 2274–78. doi: 10.1007/s00261-020-02451-4 32103300

[16] Nakajima T, Fujiwara A, Yamaguchi M, Makiyama A, Wakae T, Fujita K, et al. Intrahepatic splenosis with severe iron deposition presenting with atypical magnetic resonance images. *Intern Med* 2008; **47**: 743–46. doi: 10.2169/internalmedicine.47.0689 18421191

[17] Xuan Z, Chen J, Song P, Du Y, Wang L, Wan D, et al. Management of intrahepatic splenosis:a case report and review of the literature. *World J Surg Oncol* 28, 2018; **16**(1): 119. doi: 10.1186/s12957-018-1419-1 29954390 PMC6022698

[18] Lin W-C, Lee R-C, Chiang J-H, Wei C-J, Chu L-S, Liu R-S, et al. MR features of abdominal splenosis. *AJR Am J Roentgenol* 2003; **180**: 493–96. doi: 10.2214/ajr.180.2.1800493 12540458

[19] Marchi G, Avesani G, Zamò A, Girelli D. Unusual case of iron overload with cancer-mimicking abdominal splenosis. *BMJ Case Rep* 2018; **2018**: bcr-2017-223410. doi: 10.1136/bcr-2017-223410 PMC5965768 29769185

[20] Labranche R, Gilbert G, Cerny M, Vu K-N, Soulières D, Olivié D, et al. Liver iron quantification with mr imaging: a primer for radiologists. *Radiographics* 2018; **38**: 392–412. doi: 10.1148/rg.2018170079 29528818

[21] Venkatesh SK, Yin M, Glockner JF, Takahashi N, Araoz PA, Talwalkar JA, et al. MR elastography of liver tumors: preliminary results. *AJR Am J Roentgenol* 2008; **190**: 1534–40. doi: 10.2214/AJR.07.3123 18492904 PMC2894569

[22] Hennedige TP, Hallinan JTPD, Leung FP, Teo LLS, Iyer S, Wang G, et al. Comparison of magnetic resonance elastography and diffusion-weighted imaging for differentiating benign and malignant liver lesions. *Eur Radiol* 2016; **26**: 398–406. doi: 10.1007/s00330-015-3835-8 26032879

[23] Garteiser P, Doblas S, Van Beers BE. Magnetic resonance elastography of liver and spleen: methods and applications. *NMR Biomed* October 2018; **31**: e3891. doi: 10.1002/nbm.3891 29369503

[24] Mannelli L, Godfrey E, Joubert I, Patterson AJ, Graves MJ, Gallagher FA, et al. MR elastography: spleen stiffness measurements in healthy volunteers--preliminary experience. *AJR Am J Roentgenol* 2010; **195**: 387–92. doi: 10.2214/AJR.09.3390 20651194

[25] Talwalkar JA, Yin M, Venkatesh S, Rossman PJ, Grimm RC, Manduca A, et al. Feasibility of in vivo mr elastographic splenic stiffness measurements in the assessment of portal hypertension. *AJR Am J Roentgenol* 2009; **193**: 122–27. doi: 10.2214/AJR.07.3504 19542403 PMC2860633

[26] Xuan Z, Chen J, Song P, Du Y, Wang L, Wan D, et al. Management of intrahepatic splenosis:a case report and review of the literature. *World J Surg Oncol* 28, 2018; **16**(1): 119. doi: 10.1186/s12957-018-1419-1 29954390 PMC6022698

[27] Gandhi D, Sharma P, Garg G, Songmen S, Solanki S, Singh T. Intrahepatic splenosis demonstrated by diffusion weighted mri with histologic confirmation. *Radiol Case Rep* 2020; **15**: 602–6. doi: 10.1016/j.radcr.2020.02.022 32215161 PMC7090284

[28] Wang W-C, Li X-F, Yan Z-L, Wang Y, Ma J-Y, Shi L-H, et al. Intrahepatic splenosis mimics hepatocellular carcinoma in a patient with chronic hepatitis b: a case report and literature review. *Medicine (Baltimore)* 2017; **96**: e8680. doi: 10.1097/MD.0000000000008680 29381947 PMC5708946

[29] Inchingolo R, Peddu P, Karani J. Hepatic splenosis presenting as arterialised liver lesion in a patient with nash. *Eur Rev Med Pharmacol Sci* 2013; **17**: 2853–56. 24254551

[30] Ferraioli G, Di Sarno A, Coppola C, Giorgio A. Contrast-enhanced low-mechanical-index ultrasonography in hepatic splenosis. *J Ultrasound Med* 2006; **25**: 133–36. doi: 10.7863/jum.2006.25.1.133 16371565

[31] Grambow E, Weinrich M, Zimpfer A, Kloker K, Klar E. Ectopic spleen tissue - an underestimated differential diagnosis of a hypervascularised liver tumour. *Viszeralmedizin* 2015; **31**: 445–47. doi: 10.1159/000442115 26889148 PMC4748799

[32] Lim AKP, Patel N, Eckersley RJ, Taylor-Robinson SD, Cosgrove DO, Blomley MJK. Evidence for spleen-specific uptake of a microbubble contrast agent: a quantitative study in healthy volunteers. *Radiology* 2004; **231**: 785–88. doi: 10.1148/radiol.2313030544 15118114

[33] Gruen DR, Gollub MJ. Intrahepatic splenosis mimicking hepatic adenoma. *AJR Am J Roentgenol* 1997; **168**: 725–26. doi: 10.2214/ajr.168.3.9057523 9057523

[34] Tamm A, Decker M, Hoskinson M, Abele J, Patel V. Heat-damaged rbc scan: a case of intrahepatic splenosis. *Clin Nucl Med* 2015; **40**: 453–54. doi: 10.1097/RLU.0000000000000701 25608165

[35] Hagan I, Hopkins R, Lyburn I. Superior demonstration of splenosis by heat-denatured tc-99m red blood cell scintigraphy compared with tc-99m sulfur colloid scintigraphy. *Clin Nucl Med* 2006; **31**: 463–66. doi: 10.1097/01.rlu.0000226907.36840.b3 16855431

[36] Jolepalem P, Balon HR. Application of heat-damaged tc-99m rbcs in a patient with suspected hepatic metastasis. *Radiol Case Rep* 2013; **8**: 787. doi: 10.2484/rcr.v8i1.787 27330615 PMC4900205

[37] De Vuysere S, Van Steenbergen W, Aerts R, Van Hauwaert H, Van Beckevoort D, Van Hoe L. Intrahepatic splenosis: imaging features. *Abdom Imaging* 2000; **25**: 187–89. doi: 10.1007/s002619910042 10675464

